# CORRELATES OF FAMILY CAREGIVING FOR OLDER ADULTS AND EMOTIONAL STRAIN AMONG AFRICAN AMERICANS

**DOI:** 10.1093/geroni/igz038.1897

**Published:** 2019-11-08

**Authors:** Fei Wang, Ann W Nguyen

**Affiliations:** Jack, Joseph and Morton Mandel School of Applied Social Sciences, Case Western Reserve University, Cleveland, Ohio, United States

## Abstract

Objectives: Despite the growing older African American population and its increasing needs for informal care, few caregiving studies have focused specifically on African Americans. This study aims to 1) identify demographic correlates of caregiving for older family members among African Americans and 2) identify caregiving and demographic correlates of emotional strain among African American caregivers. Method: Logistic regression and linear regression were based on the African American sub-sample of the 2015 Caregiving in the U.S. Survey (N=260). Demographic characteristics included age, gender, education, income, marital status, co-residence of care recipient in the caregiver’s home, relationship of care recipients to caregivers, and household size. Caregiving characteristics included hours of caregiving and whether respondents provide care for an older adult. Results: With respect to demographic correlates of family caregiving, older respondents were more likely to provide care for an older family member, and respondents were more likely to provide care to a parent/parent-in-law than to other relatives. Regarding emotional strain, age and household size were negatively associated with emotional strain, and hours of caregiving was positively associated with emotional strain. Discussion: This study identified demographic profiles of family caregiving and emotional strain. It also suggested the presence of unique risk and protective factors among older African American caregivers. Future research should test the underlying mechanisms between these factors and mental health outcomes for a better understanding of how caregiving strain can be attenuated.

